# Eyelid and Vaginal Adhesions as Severe Sequelae of Toxic Epidermal Necrolysis

**DOI:** 10.7759/cureus.41496

**Published:** 2023-07-07

**Authors:** Kaori Suzuki, Yuko Watanabe, Yuichi Imai, Yukie Yamaguchi

**Affiliations:** 1 Department of Environmental-Immuno Dermatology, Yokohama City University Graduate School of Medicine, Yokohama, JPN; 2 Department of Obesterics and Gynecology, Yokohama City University Graduate School of Medicine, Yokohama, JPN

**Keywords:** hla-b*44:03, ocular sequelae, vaginal adhesion, gynecological sequelae, toxic epidermal necrolysis

## Abstract

Stevens-Johnson syndrome (SJS) and toxic epidermal necrolysis (TEN) are mucocutaneous diseases featured by severe sequelae and high mortality rates. In addition to ocular involvement, gynecological involvement is often observed in patients with TEN with possible occurrence of partial or complete adhesions of the labia majora, labia minora, and vaginal walls as severe sequelae. Although the gynecological sequelae of TEN severely affect patients’ quality of life, there is a lack of awareness among medical professionals. Moreover, preventive measures and the effectiveness of treatment have not yet been fully verified. Herein, we describe a case of TEN with severe sequelae of eyelid and vaginal adhesions.

## Introduction

Stevens-Johnson syndrome (SJS) and toxic epidermal necrolysis (TEN) are rare mucocutaneous diseases with severe sequelae and high mortality rates. An epidemiological survey in Japan revealed that approximately 97% of patients showed mucocutaneous involvements [1], particularly in the ocular region, with a high incidence of serious sequelae. The prevalence of vulvovaginal lesions in patients with SJS/TEN varies widely, estimated to be 70% in previous studies [2]. This could be even higher when patients have a chance to receive gynecological intervention. The vulvovaginal lesions in patients with SJS/TEN tend to be underrecognized. In addition, the preventive and treatment strategies are not well established. Early recognition and intervention for vulvovaginal lesions play an important role in preventing serious gynecological sequelae of SJS/TEN. Herein, we describe a patient who developed eyelid and vaginal adhesions as severe sequelae of TEN.

## Case presentation

**Figure 1:**
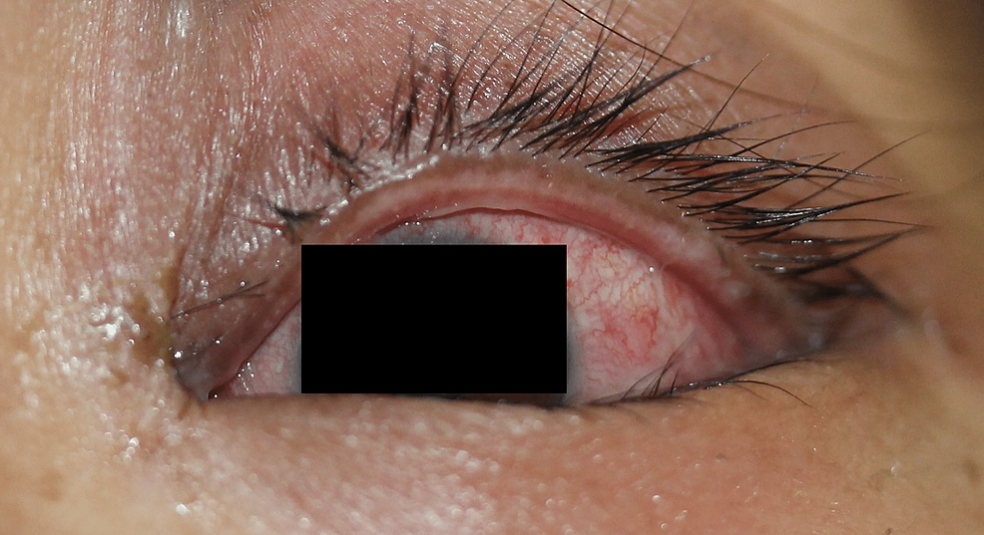
Clinical presentation of the patient - ocular conjunctival hyperemia.

**Figure 2:**
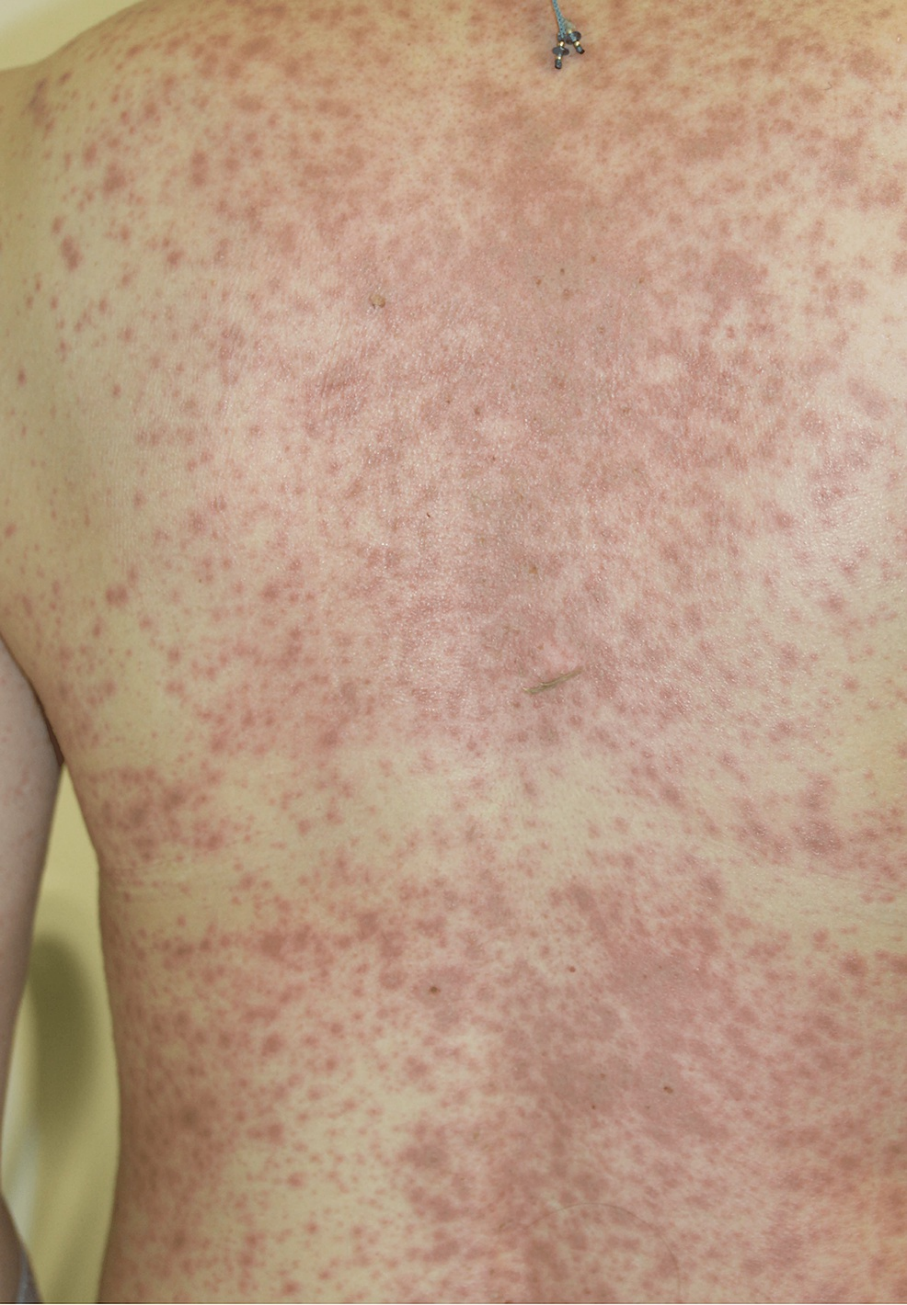
Clinical presentation of the patient - extensive erythema of the trunk.

A Japanese woman in her 40s presented with a high fever, cough, and sore throat and received acetaminophen and tranexamic acid. No other Chinese herbal medicines or supplements were administered concomitantly. One day after starting the medication, a maculopapular eruption and blisters appeared on her trunk and lips, respectively. The patient was suspected to have SJS and was transferred to our hospital. A clinical examination revealed widespread erythema on her trunk and extremities along with hyperemia of the ocular conjunctiva and hemorrhagic erosions on the lips and oral mucosa without any lesions in the genital area (Figures 1, 2).

**Figure 3:**
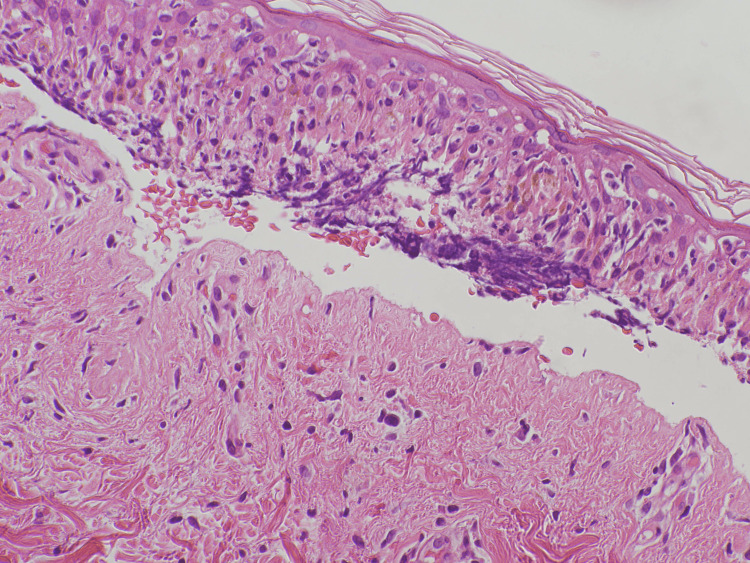
Histological findings - extensive necrosis of keratinocytes with subepidermal blister formation was observed (hematoxylin and eosin stain).

The vital signs at the initial visit were as follows: blood pressure, 80/57 mmHg; heart rate, 100/min; body temperature, 39 °C; respiratory rate, 19/min; oxygen saturation, 96% (under 1 L O2 administration). The patient’s skin tested positive for Nikolsky's sign. The laboratory findings revealed liver dysfunction (aspartate aminotransferase, 67 U/L; alanine aminotransferase, 48 U/L) and elevated C-reactive protein levels (3.92 mg/dL). The viral examination revealed a previous infection pattern with human herpesvirus antibodies. The serum *Mycoplasma pneumoniae* IgM and antigen levels were negative. The histological examination of the skin revealed full-thickness necrosis of the epidermis and subepidermal bulla (Figure 3). The area of epidermal detachment was greater than 30%, and a diagnosis of TEN was made. At that time, her severity-of-illness score for TEN (SCORTEN) was 2, predicting a mortality rate of 12.1%.

**Figure 4:**
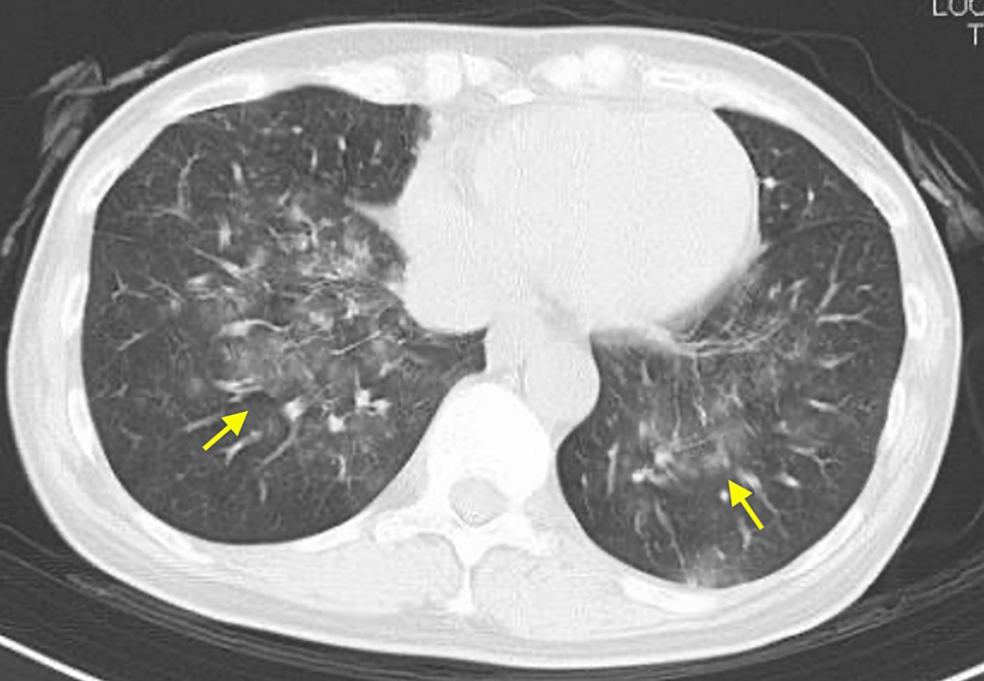
Computed tomography of the chest. Arrows indicate the spread of reticular shadows to the bilateral lungs.

**Figure 5:**
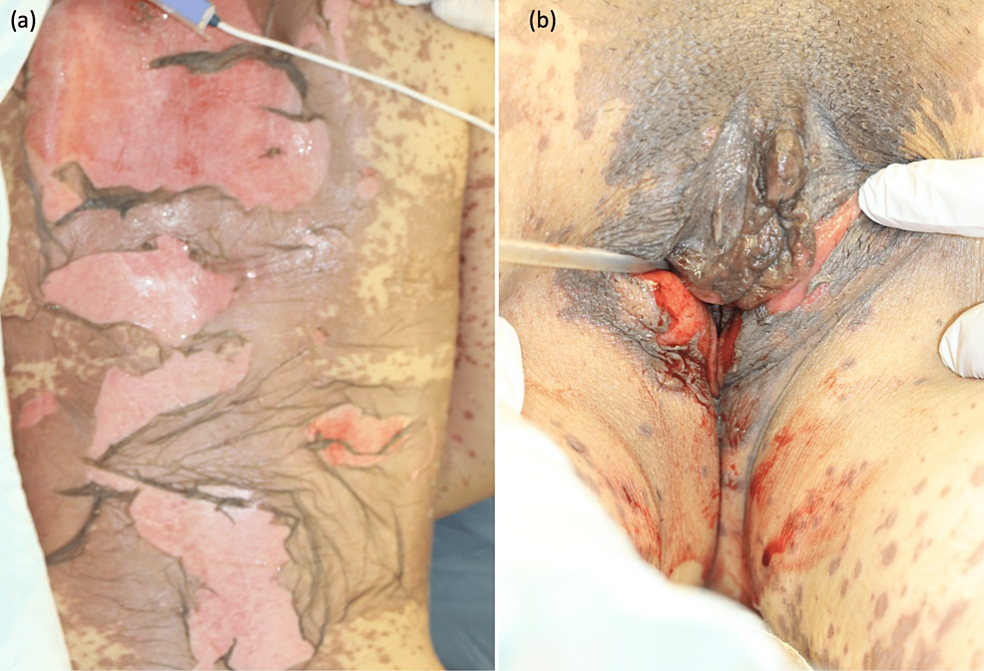
Clinical presentation during the progression of TEN. (a) Widespread epidermal detachment and (b) erosions of the genital region.

The two causative drugs, acetaminophen and tranexamic acid, were discontinued on day one. Additionally, the patient was promptly started on high-dose steroid therapy (prednisolone, 60 mg, 1 mg/kg/day). On day 2, the patient developed acute-onset dyspnea, for which mechanical ventilation was initiated. Chest computed tomography (CT) revealed bilateral spreading of reticular shadows (Figure 4). The differential diagnoses at this stage included acute respiratory distress syndrome (ARDS) induced by TEN, pulmonary edema, and coronavirus disease 2019 (COVID-19): COVID-19 testing was performed on that same day 2. Along with the rapid progression of skin erosion (approximately 60% of the body surface area), erosion and blisters appeared on the genital region (Figure 5a, 5b). Severe ocular lesions with pseudo membrane formation appeared along with a rapid expansion of skin detachment. This indicated steroid pulse therapy as a treatment option. However, it was not administered because the possibility of COVID-19 could not be ruled out at this time. Plasma exchange was performed on days 2 and 3, followed by an intravenous immunoglobulin therapy (IVIG, 400 mg/kg/day for 5 days). The ocular conditions were examined by an ophthalmologist and treated with topical steroid eye drops and ointments daily. The genital area was covered with a gauze smeared with an antibacterial ointment to prevent adhesions after the insertion of a Foley catheter. Systematic antibiotics were administered for suspected pneumonia. Thereafter, the respiratory impairments resolved promptly, both clinically and on imaging. The results of the COVID-19 polymerase chain reaction testing were negative. Rapid improvement in respiratory condition and imaging suggested that pulmonary edema could be the most suspected cause of the respiratory impairment. After these treatments, the skin gradually improved, and re-epithelization was initiated on day 8 after IVIG therapy was completed. The systemic steroids were tapered off and a complete epithelialization was observed on day 38.

**Figure 6:**
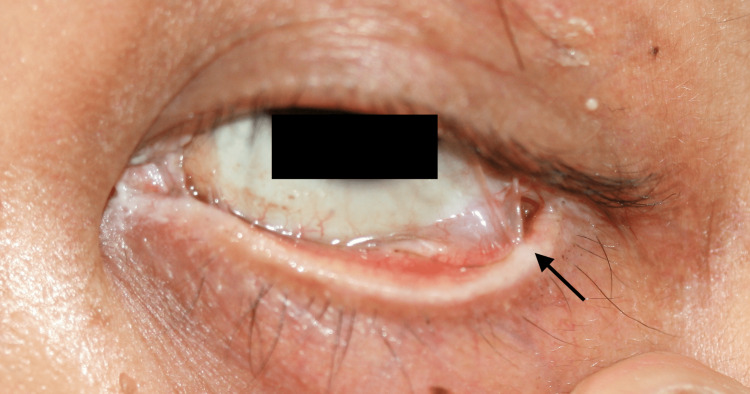
Adhesions of the eyelid as ocular sequelae. The arrow indicates eyelid adhesions.

However, a recurrence of pseudo membrane and eyelid adhesions was observed on day 6 after hospitalization, with mucous involvement resulting in eyelid adhesions, trichiasis, and severe dry eye persisted as ocular sequelae (Figure 6).

Following discharge on day 55 after hospitalization, the patient’s menstruation resumed approximately 3 months after the initial visit but was accompanied by heavy bleeding and abscess formation due to the presence of vaginal adhesions from the vulva to 2 cm above the cervix, which were difficult to dissect. Her condition improved after repeated drainage via blunt dissection and vaginal lavage. The procedure was carefully performed without sedation, as the patient experienced little or no pain. A drug lymphocyte stimulation test was performed on day 35 for the causative agents and showed negative results for tranexamic acid (394 cpm, stimulation index: 107%) and positive results for acetaminophen (860 cpm, stimulation index: 304%). The genetic testing for involvement in the development of SJS/TEN revealed that the patient had a human leukocyte antigen (HLA)-B* 44:03.

## Discussion

We encountered a case of TEN with severe ocular and gynecological sequelae in a patient with HLA-B* 44:03. Severe ocular involvement associated with SJS/TEN during the acute phase is the major cause of severe sequelae, found in approximately half of the cases [3]. The Japanese Ministry of Health, Labor, and Welfare treatment guidelines currently recommend steroid pulse therapy for progressive cases of SJS/TEN in the acute phase. In 2009, Araki et al. reported favorable systemic and visual outcomes after steroid pulse therapy without any severe infection during the treatment period [4]. Notably, early administration within 0-4 days from the onset has been reported to prevent ophthalmologic sequelae, such as the loss of corneal epithelial stem cells and severe visual impairment due to conjunctival adhesions. Approximately 80% of patients with SJS/TEN who develop severe ocular sequelae could have a history of taking cold medications following flu-like symptoms. The HLA-A*02:06 allele has been implicated in severe ocular involvement in the Japanese population, whereas the HLA-B*44:03 allele was found to be associated with these complications in the Indian, Thai, and Western Brazilian populations [5,6]. In a database on HLA allele frequency in a Japanese population of > 20,000 individuals, the prevalence of HLA-B*44-03 in the Japanese population was 6.601% [7]. In our case, the patient was one-fourth Chinese and carried the HLA-B*44:03 allele, which may have been involved in the development of TEN and its severe sequelae.

The occurrence rates of gynecological involvement were 37.5% in patients with SJS and 56.7% in those with TEN [1]. During the acute phase of SJS/TEN, partial or complete adhesions of the labia majora, labia minora, vaginal walls, and clitoral hood have been observed. In addition, the occurrence of vaginitis and blistering could lead to vaginal erosion, healing of the external genitalia, and the development of vaginal adhesions [8]. Among patients with SJS/TEN with external genital involvement, 28% were reported to have vulvovaginal sequelae [9], which are not sufficiently recognized. Moreover, no prospective clinical trials have evaluated the treatment and supportive care for gynecological complications in SJS/TEN. Furthermore, the recommendations depend on expert opinions. The goal of treatment is to preserve vaginal function and reduce adhesion formation and tumorigenic changes in tissues. Currently, the treatment options include the insertion of Foley catheters, topical corticosteroids, menstrual suppression, and physical therapy with vaginal dilators [9]. However, there are no reports on the efficacy of systemic treatments such as systemic steroids or pulse therapy. In our case, an earlier gynecologic intervention for the vulvovaginal lesions due to SJS/TEN would have been required. Topical and systemic therapy, possibly with steroid pulse therapy, may also have been appropriate to prevent severe sequelae. Further research is required to clarify this issue.

In our case, since COVID-19 was suspected, steroid pulse therapy was not administered. Thus avoiding steroid pulse therapy and the involvement of causative alleles may have contributed to the development of eyelid and vaginal adhesions as severe sequelae. It is crucial to consider gynecological interventions from the early onset of SJS/TEN to the post-healing phase, given the severe gynecological complications and sequelae.

## Conclusions

We report a case of TEN with eyelid and vaginal adhesions as severe sequelae. The avoidance of steroid pulse therapy and genetic factors may have contributed to the development of these severe sequelae. In female patients with SJS/TEN, careful attention should be paid to gynecological sequelae and early interventions should be considered. In addition, further studies on the effective treatment of gynecological sequelae are needed.
